# Updates on Children with Allergic Rhinitis and Asthma during the COVID-19 Outbreak

**DOI:** 10.3390/jcm10112278

**Published:** 2021-05-24

**Authors:** Giulia Brindisi, Valentina De Vittori, Rosalba De Nola, Elia Pignataro, Caterina Anania, Giovanna De Castro, Bianca Cinicola, Alessandra Gori, Ettore Cicinelli, Anna Maria Zicari

**Affiliations:** 1 Department of Maternal Infantile and Urological Sciences, Division of Pediatric Allergology and Immunology, Sapienza University of Rome, 00185 Rome, Italy; valentinadevittori@gmail.com (V.D.V.); e.pigna91@gmail.com (E.P.); caterina.anania@uniroma1.it (C.A.); giovanna.decastro@uniroma1.it (G.D.C.); biancacinicola@gmail.com (B.C.); alessandra.gori85@gmail.com (A.G.); annamaria.zicari@uniroma1.it (A.M.Z.); 2 Department of Biomedical Science and Human Oncology, Gynecology and Obstetrics Section, University of Bari “Aldo Moro”, Piazza Giulio Cesare 11, 70124 Bari, Italy; denolarosalba@gmail.com (R.D.N.); ettore.cicinelli@uniba.it (E.C.); 3 Department of Tissues and Organs Transplantation and Cellular Therapies, University of Bari “Aldo Moro”, Piazza G. Cesare 11, 70124 Bari, Italy

**Keywords:** COVID-19, SARS-CoV-2, lockdown, children, asthma, rhinitis, allergy

## Abstract

Background: During the lockdown period caused by the SARS-CoV-2 pandemic, we monitored via online survey the trend of allergic symptoms and the therapeutic compliance in patients followed at our center. Material and methods: In June 2020, we selected children followed at the Allergy and Immunology Service of Umberto I Hospital, aged between 6 and 16 years old, diagnosed with asthma and/or rhinitis and sensitized to grass pollen or dust mite. We sent an email with 12 multiple-choice questions investigating several areas: type of disease and sensitization, recurrence of symptoms, medication use during lockdown compared to the same period of the previous year. Results: The results of 82 questionnaires showed that 17.8% of patients suffered from asthma, 24.4% from rhinitis, and 57.8% from both. Within the group of asthmatic children, most of them presented an improvement of their symptoms. Likewise, with regard to allergic rhinitis, most of them reported better clinical conditions. Regarding treatment, we observed a global decrease in the use of on-demand therapies (salbutamol, nasal corticosteroid, and antihistamine) for both pathologies. In addition, there was a reduction in the use of basal therapy for asthma and rhinitis from 2019 (23.3%) to 2020 (15.5%). Conclusions: Our data show a general trend of clinical improvement and a reduction in the use of on-demand and basal therapy in allergic children during the lockdown.

## 1. Introduction

Since December 2019, a new coronavirus, SARS-CoV-2, has spread worldwide; on March 2020, the World Health Organization (WHO) declared a global pandemic [1]. At the time of writing this paper (April 2021), there have been about 135,446,538 confirmed SARS-CoV-2 cases worldwide. Of all these cases, 3,668,264 were reported in Italy, one of the most affected countries, which has counted 111,070 deaths so far [2]. The Italian National Institute of Health gives regular updates with detailed data [3]. From recent reports, Italian pediatric cases (between 0 and 19 years of age) amounted to 518,057, corresponding to 14.1% of all the affected population, with 22 pediatric deaths. SARS-CoV-2 infection in children is less common, often spreads within family clusters, and manifests with mild and varied symptoms [4,5] such as fever, nasal congestion, cough, dyspnea, myalgia, arthralgia, headache, gastrointestinal, and skin manifestations with the characteristic anosmia and ageusia [6,7]. Rare cases have shown severe respiratory symptoms requiring intensive care or a multisystem inflammatory syndrome (MIS-C) [8]. Regarding all the susceptibility factors associated with SARS-CoV-2 infection, Du H et al. studied a cohort of 182 children suffering from COVID-19 and did not report any difference between allergic and non-allergic children, arguing that allergy is not a risk factor for SARS-CoV-2 infection [9]. Furthermore, it has been hypothesized that allergy could be a protective condition [10]. Indeed, the presence of eosinophils would correlate with a reduced expression of the angiotensin conversion enzyme receptor 2 (ACE2), the entrance door of the SARS-CoV-2 into the cells of the respiratory tract [11].

However, uncontrolled asthma symptoms can represent a risk factor for the severity of SARS-COV-2 infection. Thus, the goal was to control the symptoms of allergic diseases in the daily clinical practice and also during the SARS-CoV-2 infection [12,13].

This survey aimed to monitor, through an online questionnaire, the impact of lockdown on the allergic symptoms and the use of medications in a group of children sensitized to grass pollen and dust mite, in comparison to the same period of the previous year.

## 2. Materials and Methods

### 2.1. Study Design and Population

This is a retrospective study. In June 2020, we selected children aged between 6 and 16, sensitized to grass pollen, dust mite, or both, with a diagnosis of asthma and/or rhinitis, followed at the Department of Pediatric Allergy and Immunology of Umberto I Hospital in Rome. Exclusion criteria were chronic respiratory, cardiac, and immunologic conditions, and a known poor compliance of the patients’ caregivers.

We sent a questionnaire by email to the parents of all enrolled children. The questionnaire contained 12 multiple choice questions about the allergy of their children during the lockdown period (from the beginning of March to the beginning of June 2020). The questionnaire took approximately 10 min to answer. We sent 120 questionnaires and received 82 responses. Through Google Forms, anonymous data were automatically stored in an Excel format, useful for statistical analysis. The questionnaire (reported in the Data S1) investigated several areas:

− Age and gender of the patient;
− Data of medical history: type of disease (asthma, rhinitis, or both) and sensitization (dust mite, grass pollen, or both);
− Worsening or improvement of allergic symptoms during lockdown compared to the same period of the previous year;
− Use of preventive drugs for asthma and rhinitis (inhaling or nasal corticosteroids, long-acting bronchodilators) or on-demand treatment (nasal corticosteroids, antihistamines, bronchodilators) during the lockdown, compared to the same period of the previous year.

The Ethics Committee of Sapienza University of Rome, did not consider any special permission necessary because the study’s design met the criteria of activity audit. Informed consent was not obtained, because the participation was voluntary.

### 2.2. Statistical Analysis

All the variables were completed. Except for the age variable (numeric), all the variables were factors. Statistical analyses were performed using the R statistical environment (The R Foundation for Statistical Computing; Vienna, Austria), specifically the packages “fBasics”, “graphics”, “ggplot2”, “lawstat”, “gmodels”, “pwr”, and “psych”. The Shapiro–Wilk test and graphical evaluations of the numerical variable were performed to demonstrate the correspondence with the normal distribution. Therefore, the age was approximated as normally distributed. The Bartlett test was performed to evaluate the homogeneity of age’ variances: the variable was approximated as homoscedastic. A modified robust Brown–Forsythe Levene-type test, based on the absolute deviations from the median, confirmed the Bartlett test’s results. Therefore, the age was considered normal and homoscedastic. The categorical variables were illustrated as frequencies (%), whereas the descriptive statistical data were reported as mean ± standard deviation. Since the variable age was approximately normal and homoscedastic, we used the one-way analysis of variance (ANOVA) to compare differences between groups. The relationship between categorical factors was evaluated using the chi-squared test and Fisher’s Exact Test (package “gmodels”). However, the latter was preferred due to the sample size. Plots and graphs were realized using the R package “graphics”, “vcd”, and “ggplot2”. A two-sided *p*-value < 0.05 was considered to indicate statistical significance in respect to a medium effect size (0.3).

## 3. Results

We enrolled 82 patients, 57 (69%) were males and 25 (31%) were females, with a mean age of 9.7 ± 3.06 years old. 

Among all the examined children, 17.8% suffered from asthma, 24.4% from rhinitis, and 57.8% from both asthma and rhinitis. Regarding sensitization, 36.7% were allergic to dust mite, 22.2% to grass pollen, and 41.1% were allergic to both dust mite and grass pollen.

**Figure 1:**
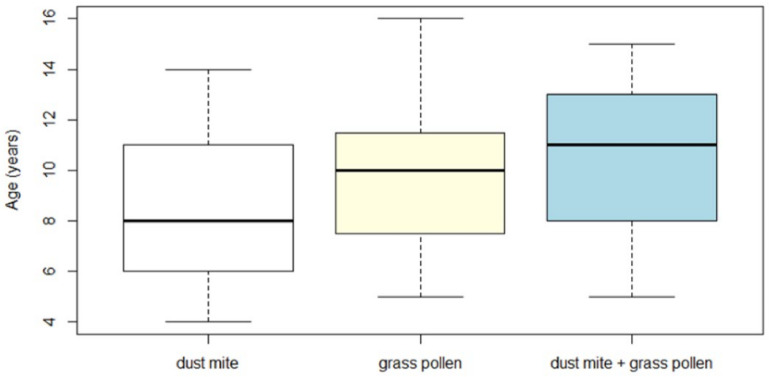
Age distribution of the enrolled children depending on the type of allergy (dust mite, grass pollen, dust mite + grass pollen).

With regard to the age, we found a significant difference (*p* < 0.05) among children sensitized to dust mite, grass pollen, or both. Children allergic to dust mite were younger (8.7 ± 3 years) than those allergic to grass pollen (9.8 ± 2.9 years) and those allergic to both allergens (10.5 ± 3.01 years) (Figure 1).

More specifically, the age of the dust mite group was significantly lower than the age of the polysensitized group (dust mite + grass pollen), with a *p*-value < 0.05 for Bonferroni and *t*-Student. The other two comparisons (grass pollen vs. dust mite and grass pollen vs. dust mite + grass pollen) had a non-significant difference (Bonferroni and *t*-Student, *p* > 0.05). Under a high-power (0.8), the effect size of the ANOVA test was medium (0.3). 

The box plot of the age distribution of the enrolled children depended on the type of allergy: children allergic to dust mite had a lower median age (8.7 ± 3 years), followed by those allergic to grass pollen (9.8 ± 2.9 years), and those poliallergic (10.5 ± 3.01 years).

Compared to the same period in 2019, within the group of children with asthma 64.7% presented an improvement of symptoms, 27.9% did not show any change, and only 7.3% reported a worsening of their condition. To be more precise, within the asthma group with an improvement of symptoms, 29% of children were allergic to dust mite, 32% to grass pollen, and 39% to both allergens. However, analyzing the asthma group with a worsening of symptoms, 40% of children were allergic to dust mite, 40% to dust mite + grass pollen, and 20% to grass pollen.

Regarding the group of children with rhinitis, 48.6% presented an improvement of their symptoms, 44.5% did not report any change, and 6.7% presented a worsening of rhinitis.

In the rhinitis group with improved symptoms, 52.7% of children were polysensitized, 30.55% were allergic to grass pollen, and 16.66% to dust mite. The children who experienced a worsening of their symptoms were mainly polisensitized (60%), whereas the other groups were in equal percentages (20%).

**Figure 2:**
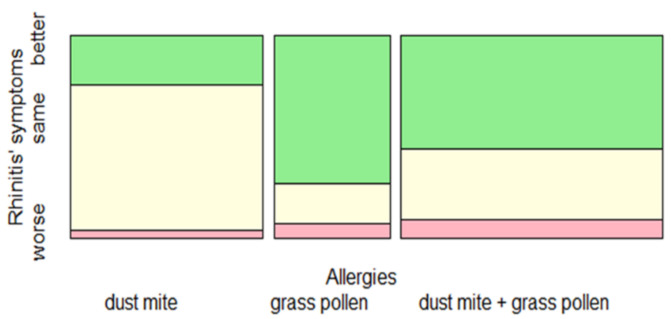
Change in rhinitis symptoms during the lockdown (worse, same, better), depending on allergic sensitization.

Fisher’s Exact Test was performed between the three types of allergies (dust mite, grass pollen, dust mite + grass pollen) and the entity of rhinitis symptoms (worse, same, better). It showed significant (*p* < 0.05, effect size 0.3, power 0.39) differences among them. More specifically, patients allergic to grass pollen, followed by those polysensitized (dust must + grass pollen), presented a significantly higher frequency of an improvement of rhinitis symptoms during home quarantine compared to those allergic only to dust mite (Figure 2).

Spineplot represented the distribution of rhinitis symptoms during the lockdown as related to allergic sensitization. Children allergic to grass pollen followed by those polysensitized had a significant improvement of rhinitis symptoms compared to children allergic to dust mite (*p* < 0.05, effect size 0.3, power 0.39). 

With regard to on-demand therapy used during lockdown, we observed that 94.1% of children with asthma required less use of salbutamol, 5.8% required the same use, while no one needed to use more of this drug compared to the previous year. Indeed, among patients with rhinitis, we observed that 51.3% reduced the use of nasal corticosteroid, 36.4% required the same use, and 12.1% used more nasal therapy than the previous year.

Regarding oral antihistamines, 47.8% of all the enrolled patients used it less frequently, 32.6% reported the same use, and 19.6% reported a more frequent use than the previous year. 

We also registered a reduction of asthma and rhinitis basal therapy from spring 2019 (23.3%) to spring 2020 (15.5%). 

## 4. Discussion

In the lockdown era, respiratory diseases such as asthma required less hospitalization than the previous years due to a reduction in respiratory tract infections [14,15]. Different reasons can explain these results, such as public health interventions during the COVID-19 pandemic and the consequent reduction of exposure to the exacerbating factors for allergy [16].

So far, only few studies in pediatric populations have shown the effects of the lockdown on allergic symptoms. We used an online survey to monitor symptoms and therapy in our cohort of allergic patients during the pandemic. Most patients reported an improvement of their asthma and rhinitis symptoms in respect to the previous year. Only a small percentage reported worse clinical conditions, in agreement with the international literature. The reduction of allergic symptoms can be found primarily in the grass-pollen group for several reasons, such as a reduced pollen exposure and a better and prompt management of symptoms by parents more present at home. Moreover, during the lockdown, there was a reduction of air pollution, a well-known risk factor for allergic diseases, and a remarkable reduction of respiratory viruses [17,18]. Instead, we found that the group that improved the least was the dust-mite group, maybe due to a persistent exposure to this allergen in the domestic environment during the lockdown. Other studies are in line with our findings. In adults, Gelardi et al. conducted a tele-health consultation on 45 patients allergic to dust mite, submitting a questionnaire with the sinosal outcome test (SNOT-22). They concluded that the lockdown has negatively influenced patients’ clinical symptoms with allergic rhinitis sensitized to dust mite, suggesting that being quarantined at home increased the exposure to indoor allergens [19].

Furthermore, Gallo et al. underlined that in adults, the COVID-19 lockdown may have ameliorated the symptoms and quality of life in seasonal allergic patients but worsened allergic symptoms in those sensitized to dust mite [20].

The current guidelines for allergic children in the COVID-19 period consider it appropriate to continue therapy for rhinitis and asthma control. The interruption of the treatment can lead to a lack of control and an increased risk of exacerbations [21,22]. Allergic patients with an acute phase of COVID-19 infection should continue standard therapy, except biological drugs and allergen immunotherapy (AIT). They should use on-demand therapy during exacerbations [23,24]. According to the improvement of symptoms in our patients, the use of on-demand therapy (salbutamol for asthma and nasal steroids/antihistamine for rhinitis) and basal therapy was markedly lower during the lockdown compared to the same period in 2019.

This survey presents some limitations. First of all, this study was conducted on a small number of patients recruited in the same outpatient setting. Furthermore, it was conducted using a non-standardized questionnaire filled out by parents at home, without medical supervision. These can lead to some bias toward over or underestimation. 

## 5. Conclusions

In conclusion, this is the first Italian survey conducted in a pediatric population with allergic rhinitis and/or asthma to detect changes in symptoms and use of medications during the COVID-19 lockdown. Our data show a general trend of clinical improvement and a reduction of on-demand and basal therapy. Possible explanations are the presence of parents at home with an increased cleaning of dust mite, the better management of allergic symptoms, the less frequent exposure to grass pollens and viruses, and a general reduction in air pollution. 

## Data Availability

The data presented and analysed during the current study are available on request from the corresponding author.

## Supplementary Materials

The following are available online at https://www.mdpi.com/article/10.3390/jcm10112278/s1, Data S1: COVID-19 and allergic asthma and Rhinitis Questionnaire.
