# Supplementary material for: Updates on Children with Allergic Rhinitis and Asthma during the COVID-19 Outbreak
Source: J Clin Med. 2021 May 24;10(11):2278. doi: 10.3390/jcm10112278 (PMC8197398; doi:10.3390/jcm10112278)
Supplement: Supplementary file 1 [file jcm-10-02278-s001.zip › jcm-1200838-supplementary.pdf]

Supplementary Material

COVID-19 and allergic asthma and Rhinitis Questionnaire

1. Gender of the patient:

-Female

-Male

2. Age of the patient (years): ……………

3.Your child is affected by:

- Rhinitis
- Asthma
- Both

4. Your child is allergic to:

- Dust mite
- Grass Pollen
- Alternaria
- Other pollens

5. During home quarantine (March/June 2020), compared to the same period of the previous year, your child’s rhinitis symptoms are:

- Worsened
- Same
- Improved

6. During home quarantine (March/June 2020), compared to the same pertiod of the previous year, your child's asthma symptoms are:

- Worsened
- Same
- Improved

7. In case of improvement or worsening of rhinitis symptoms, express the degree:

- No improvement/worsening
- Slight improvement/worsening
- Moderate improvement/worsening
- Consistent improvement/worsening

8. In case of improvement or worsening of asthmatic symptoms, express the degree:

-No improvement/worsening

-Slight improvement/worsening

-Moderate improvement/worsening

-Consistent improvement/worsening

9. Your child performed basal therapy with corticosteroid for asthma and/or allergic rhinitis:

- This year
- Last year
- Both

10. Compared to the previous year, your child used on demand therapy for asthma exacerbations:

-Not used

-With the same frequency

-More frequently

-Less frequently

11. Compared to the previous year, your child used nasal corticosteroid spray for rhinitis flare-ups:

-Not used

-With the same frequency

-More frequently

-Less frequently

12. Compared to the previous year, your child used oral antihistamines:

-Not used

-With the same frequency

-More frequently

-Less frequently
